# Identification and splicing analysis of the first deep intronic *FIG4* variant causing Yunis–Varon syndrome

**DOI:** 10.3389/fgene.2025.1624122

**Published:** 2025-08-08

**Authors:** Hui Tang, Qingqing Chen, Jingjing Xiang, Qin Zhang

**Affiliations:** Center for Reproduction and Genetics, School of Gusu, The Affiliated Suzhou Hospital of Nanjing Medical University, Suzhou Municipal Hospital, Suzhou, Jiangsu, China

**Keywords:** Yunis–Varón syndrome, *FIG4* gene, deep intronic variant, whole-genome sequencing, prenatal diagnosis

## Abstract

Yunis–Varón syndrome (YVS) is a severe autosomal recessive syndrome caused by mutations in the *FIG4* gene. It is characterized by skeletal defects, including cleidocranial dysplasia and digital anomalies, and a poor prognosis due to neurological and cardiovascular involvement. In this study, we observed a Chinese family with three patients presenting thumb and hallux dysplasia. Whole-genome sequencing (WGS) identified a compound heterozygous variant in the proband: c.2097-809A>G and c.1141C>T (p.R381*). The c.2097-809A>G variant generated an aberrant splicing transcript containing a pseudoexon from intron 18, as demonstrated by further RT-PCR and splicing analysis. This is the first deep intronic variant reported in the *FIG4* gene. In addition, we provided prenatal diagnoses for the family. This study expands the genetic variant spectrum, provides additional molecular and clinical information, and broadens our understanding of the molecular mechanisms involved in the disease course.

## 1 Introduction

Phosphatidylinositol 3,5-bisphosphate (PI(3,5)P2) is one of the seven known phosphoinositides involved in the orchestration of vesicle trafficking in mammalian cells (Martyn and Li, 2013). The *FIG4* gene encodes a phosphoinositide 5-phosphatase that regulates the synthesis and turnover of PI(3,5)P2 and is involved in endolysosomal trafficking (Sbrissa et al., 2007; Martyn and Li, 2013; Lees et al., 2020). Variants in *FIG4* can cause four different diseases: bilateral temporo-occipital polymicrogyria (OMIM 612691), amyotrophic lateral sclerosis 11 (OMIM 612577), Charcot–Marie–Tooth disease, type 4J (CMT4J) (OMIM 611228), and Yunis–Varón syndrome (YVS) (OMIM 216340). In an animal model, homozygous *FIG4*-null mice exhibit neurological features of YVS, including degeneration of the brain and peripheral ganglia and enlarged vacuoles in neurons; they also present skeletal deformities, with reduced trabecular bone volume and cortical thickness, and accumulate large vacuoles (Chow et al., 2007; Ferguson et al., 2012; Campeau et al., 2013).

Yunis–Varón syndrome is a very rare autosomal recessive syndrome that exhibits severe skeletal abnormalities (cleidocranial dysplasia, absence/hypoplasia of thumbs and halluces, pelvic bone dysplasia, and fractures), which are often lethal in infancy (Yunis and Varon, 1980; Basel-Vanagaite et al., 2008). In addition, neurological features such as hypotonia, dysphagia, global developmental delay, brain malformations, retinopathy, and facial dysmorphisms have also been attributed to it (Basel-Vanagaite et al., 2008; Campeau et al., 2013). By describing and reviewing the literature, some studies of *FIG4*-associated YVS have been reported (Reutter et al., 2012; Nakajima et al., 2013; Peng et al., 2021; Umair et al., 2021; Wang et al., 2023; Beauregard-Lacroix et al., 2024). However, only a few of them carried out appropriate genetic analysis and diagnosis. Variant analysis of the *FIG4* gene is a valuable tool for screening and diagnosis of YVS. So far, we have found 18 variants of YVS that have been recorded: c.350C>T, c.2376 + 1G>T (Wang et al., 2023), c.573del, c.2174dup (Peng et al., 2021), c.1260_1261del, c.311G>A, c.831_838del, c.524T>C (Campeau et al., 2013), c.968A>G (Umair et al., 2021), c.1750+1del, c.2285_2286del (Nakajima et al., 2013), c.1141C>T, c.2459+1G>A, c.1294C>T, c.122T>C, c.1474C>T, c.2285_2286del, and c.1583+1G>T (Beauregard-Lacroix et al., 2024). All of them have severe clinical features of YVS, such as skeletal abnormalities, craniofacial dysmorphisms, and structural brain anomalies. We also observed prenatal phenotypes in the fetuses.

In this study, we report the identification of a novel deep intronic *FIG4* variant (c.2097-809A>G) in a Chinese family with three neonates presenting hallmark features of YVS. The c.2097-809A>G variant, the first deep intronic variant reported in the *FIG4* gene, was identified by whole-genome sequencing (WGS) and confirmed by Sanger sequencing. Further splicing analysis of RNA from peripheral blood mononuclear cells (PBMCs) demonstrated that c.2097-809A>G generated an aberrant transcript that destroyed the function of *FIG4*. Therefore, we provided prenatal diagnosis for this family. This study expands the genetic variant spectrum, provides additional molecular and clinical information, and broadens our understanding of the molecular mechanisms involved in the disease course.

## 2 Methods

### 2.1 Participants and ethical statement

This study included three children from a Chinese family who received genetic counseling at Suzhou Municipal Hospital in 2022. Written informed consent was obtained from the patients’ parents, and all procedures were conducted in accordance with the tenets of the Declaration of Helsinki. The research was performed under the approval of the Ethics Committee of Suzhou Municipal Hospital, the Affiliated Suzhou Hospital of Nanjing Medical University (approval no. 2017005).

### 2.2 DNA and RNA isolation

We obtained blood samples from a child, his parents, and normal controls and the amniotic fluid from the fetus. A younger afflicted child died soon after birth, and we could not collect his samples. Genomic DNA was extracted using the QIAamp DNA Blood Mini Kit (QIAGEN, Hilden, Germany), according to the standard extraction methods. Total RNA from the blood leukocytes of the patients and controls was extracted using the standard TRIzol method. cDNAs were synthesized from RNA using the RevertAid First Strand cDNA Synthesis Kit (Thermo Fisher Scientific, Waltham, United States).

### 2.3 Whole-genome sequencing

Qualified genomic DNA was randomly fragmented into proper pieces (350 bp). WGS libraries were prepared using the MGIEasy FS DNA Prep kit (BGI, Beijing, China), according to the manufacturer’s instructions. Then, they were sequenced using DNBSEQ-T7 (MGI, Beijing, China). The minimal read depth was 30-fold for WGS. Quality control for the paired-end reads was assessed using FastQC (https://www.bioinformatics.babraham.ac.uk/projects/fastqc/), and fastp (Chen et al., 2018) was used for adapter trimming and quality filtering.

### 2.4 Variant analysis by direct sequencing

Direct Sanger sequencing in both directions was conducted to validate putative variants. The sequence containing the variant was amplified by polymerase chain reaction (PCR) with the primers *FIG4* exon 11 (forward, 5′- GGA​TTT​GAA​CCC​AGG​CAG​TAC​T-3′ and reverse, 5′-TCG​CTG​ATC​TCC​CAA​AAC​TTA​CT-3′) and *FIG4* intron 18 (forward, 5′- ACT​GTT​AGT​GGT​TTT​GGT​CCT-3′ and reverse, 5′-TGT​TGA​TTT​CTG​CTC​AGG​GA-3′). PCR products were verified by agarose gel electrophoresis and subsequently sequenced in both directions using an ABI 3130 Genetic Analyzer (Applied Biosystems, United States).

### 2.5 Reverse transcription polymerase chain reaction and TA cloning

After extracting total RNA from the neonate and healthy controls, cDNAs were synthesized from RNA using the RevertAid First Strand cDNA Synthesis Kit (Thermo Fisher Scientific, United States). DNA fragments of *FIG4* intron 18 were amplified with the following primers: forward, 5′- TCC​TGG​GAG​TTT​TCC​ATC​CCA​CT-3′ and reverse, 5′-CTG​GCT​GCC​GTT​TTC​CGC​TG-3′. The expected PCR product size was 451 bp. TA cloning was performed to isolate the aberrant transcript. PCR amplification was ligated to the T-Vector pMD19 (Simple) (TaKaRa, Japan). The positive clones were selected by blue/white screening with β-gal selection, and plasmids were extracted from the enriched white isolations using the Plasmid Mini Kit (QIAGEN, Hilden, Germany). For each sample, 10 bacterial clones were sequenced. The sequence of the amplified segment was compared with the reference sequence at UCSC.

### 2.6 Prenatal diagnosis

Following the identification and analysis of the *FIG4* variant in the family members, prenatal genetic testing was performed for the fetus. In brief, an amniotic fluid sample was collected under ultrasonic guidance from the proband at 22 weeks of gestation. DNA was extracted using a DNA extraction kit (TIANGEN Biotech, Beijing, China), according to the manufacturer’s instructions. The *FIG4* gene variant was detected using the same methods as previously described.

## 3 Results

### 3.1 Clinical features

**FIGURE 1:**
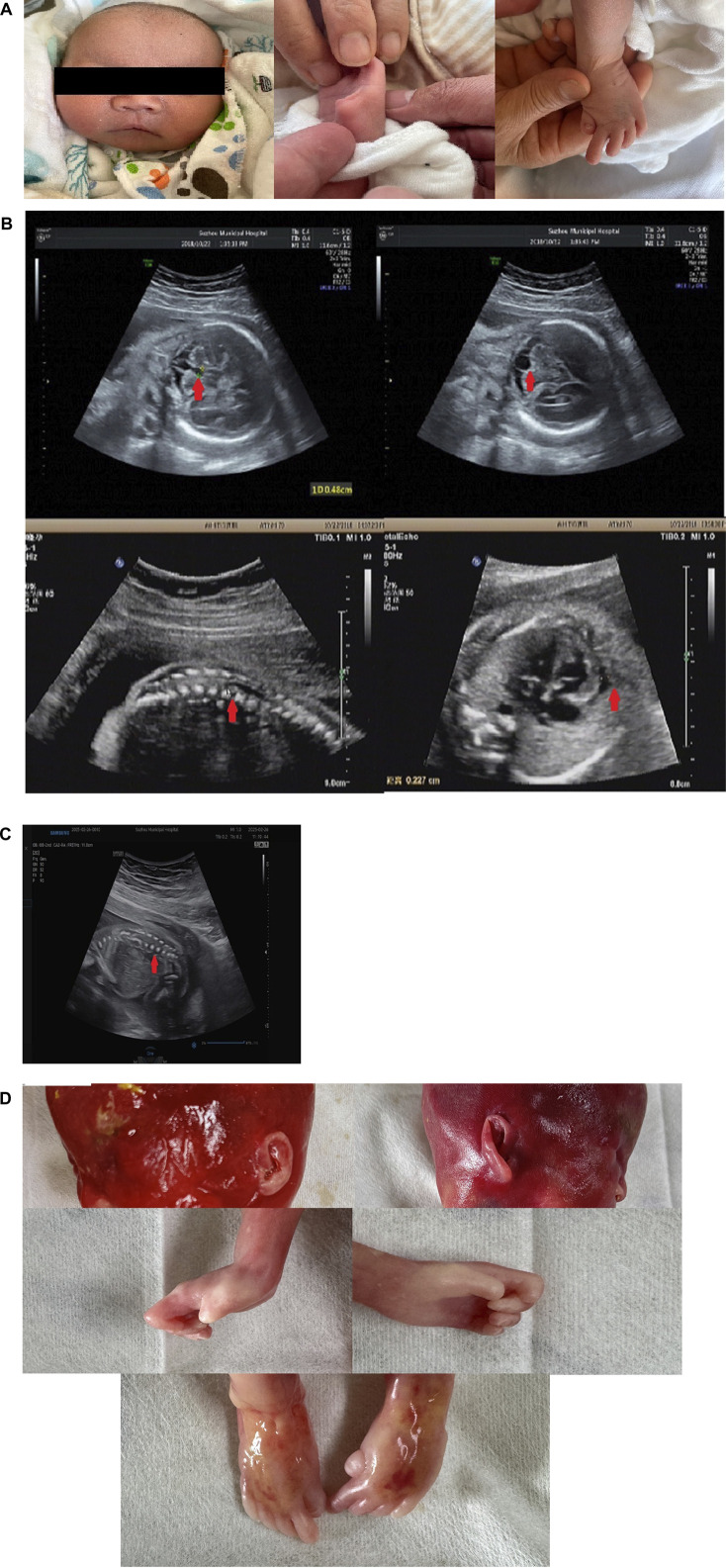
Phenotype of the patients: **(A)** facial malformation and thumb and toe anomalies of the proband. **(B)** Ultrasound scan of II-1 showed a dilated fourth ventricle, a posterior fossa cyst, a low conus medullaris position, and pericardial effusion. **(C)** Ultrasound scan of II-3 showed low conus medullaris position. **(D)** Low ear position and dysplasia of thumbs and halluces in II-3.

**FIGURE 2:**
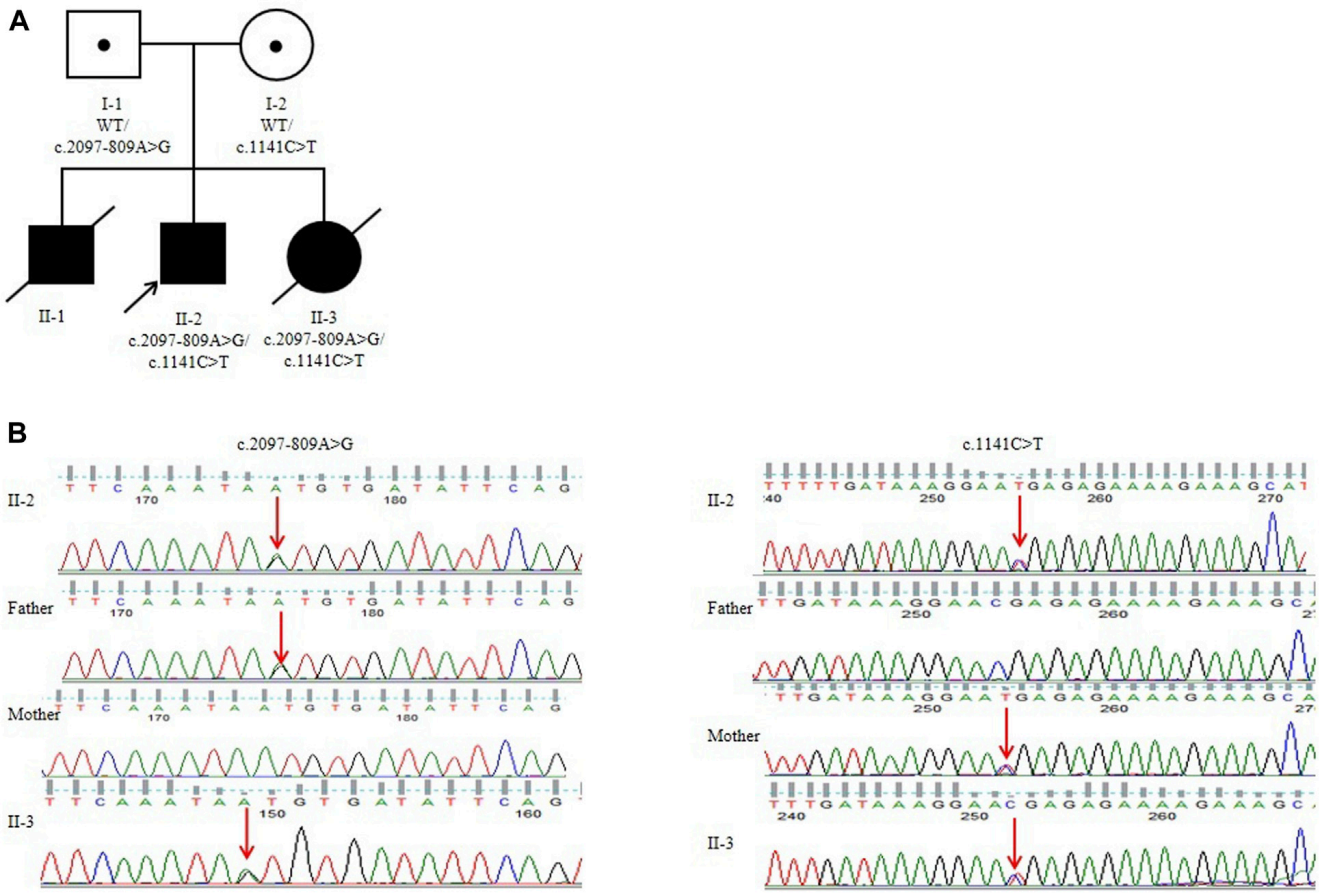
Pedigree analysis of the Chinese family: **(A)** genomic sequencing of the proband and his family. **(B)** I-1: Father of the proband, who carried a heterozygous variant of c.2097-809A>G; I-2: mother of the proband, who carried a heterozygous variant of c.1141C>T; II-2: the proband in this family, who carried two compound heterozygous variants of c.2097-809A>G and c.1141C>T. II-3 carried two compound heterozygous variants of c.2097-809A>G and c.1141C>T.

The patient (II-2) was a 16-day-old newborn at the time of genetic consultation in 2022. He was born through cesarean section with mild asphyxia, weak crying, dysphagia, feeding difficulties, and neonatal hypotonia. He exhibited multiple facial deformities, such as a broad forehead, long philtrum, hypertelorism, depressed nasal bridge, low ear position, retrognathia, short neck, and yellow hair. His bilateral, extremely short thumbs and big toes are notable characteristics of YVS (Figure 1A). He also developed hypospadias. The echocardiography also showed an atrial septal defect and atrial septal aneurysm. His mother had an earlier child in 2019 (II-1) with similar defects. The 24-week ultrasound results showed multiple malformations, including a dilated fourth ventricle, posterior fossa cyst, low conus medullaris position with coccyx curvature abnormality, persistent left superior vena cava, slightly enlarged right heart, and a small amount of pericardial effusion (2.3 mm) (Figure 1B). After birth, he presented with abnormal skull morphology, metopic synostosis, and dyspnea. His hypoplastic thumbs and big toes were identical to those of II-2, and he died shortly thereafter. The parents denied consanguinity and any family history of related conditions (Figure 2A).

### 3.2 Identification of the variant

To examine the variant, we performed WGS using DNA from the proband and his parents’ PBMCs. The average depth of WGS was 30×, and the percentage of coverage over 10× was 96.4%, meeting the qualification for subsequent analysis. After filtering the variants using the minor allele frequency (MAF) in the 1000 Genomes Project and the Genome Aggregation Database (gnomAD) and searching for suspicious variants by the characteristic phenotype, c.1141C>T(p.R381*) in *FIG4* was identified as being inherited from the mother. c.1141C>T (p.R381*) has been reported in two children with early-onset CMT4J (Nicholson et al., 2011; Zimmermann et al., 2020). In addition, the clinical findings of the two patients were highly suggestive of YVS caused by pathogenic variants in *FIG4*. Next, an in-depth search and analysis were performed. Then, we found c.2097-809A>G in the proband, which was inherited from the father. c.2097-809A>G was absent in various genetic variant databases or websites such as the ClinVar Database, Human Gene Variant Database (HGMD), Leiden Open Variation Database (LOVD), and PubMed. The online website SpliceAI predicted that it would destroy the acceptor sites with a score of 0.97. Subsequent family genotyping from his parents confirmed the compound heterozygous forms (Figure 2B).

### 3.3 RT-PCR and splicing analysis

**FIGURE 3:**
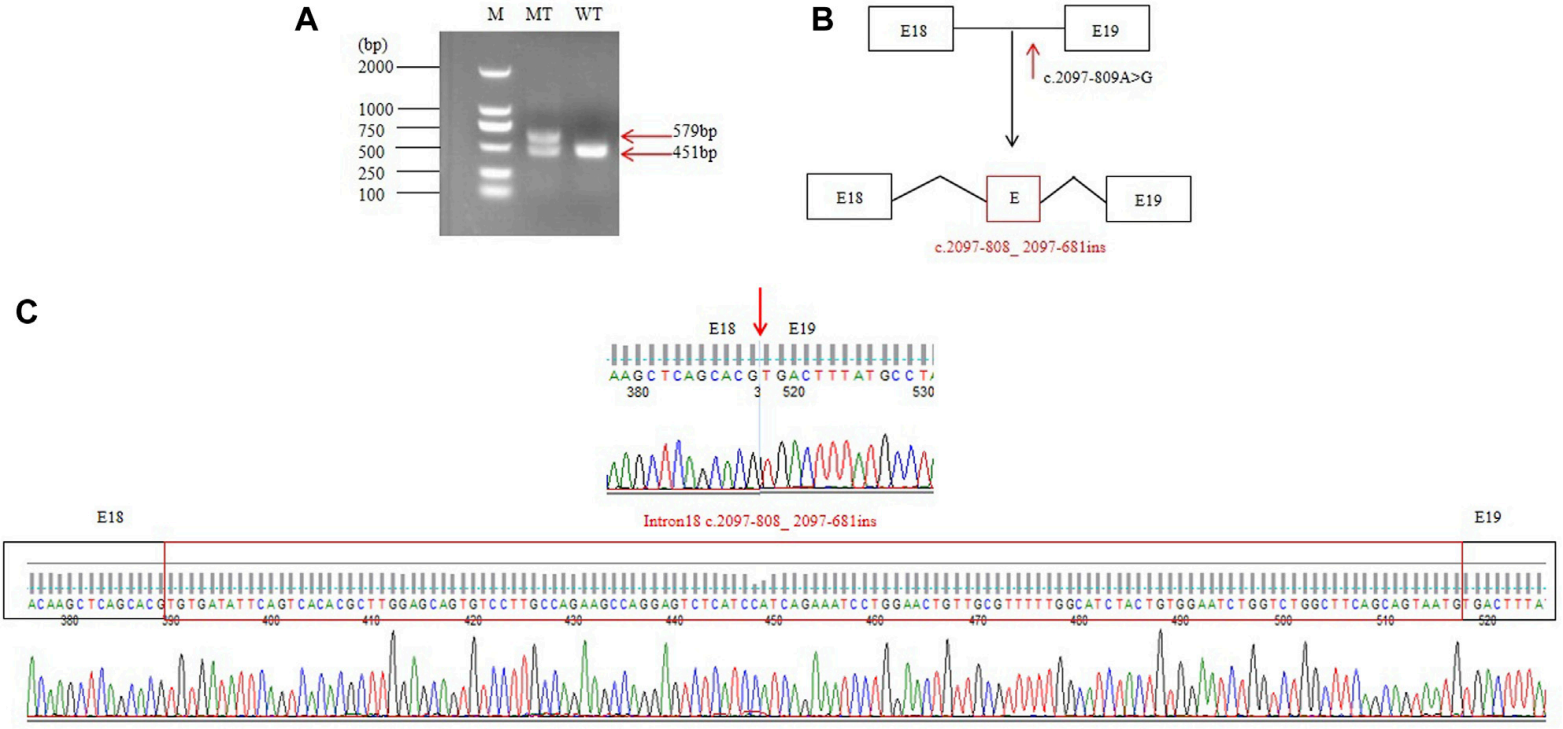
RT-PCR and splicing analysis of the c.2097-809A>G: **(A)** reverse transcription PCR analysis of mRNAs from peripheral blood leukocytes of one normal control and the patient. WT exhibited one normal splicing product (451 bp), and the patient showed a major aberrant transcript (579 bp). **(B)** Schematic of normal and aberrant splicing patterns. **(C)** Sanger sequencing of the full-length 451-bp transcript and the 579-bp aberrant transcript that included a 128-nt pseudo-exon.

**FIGURE 4:**
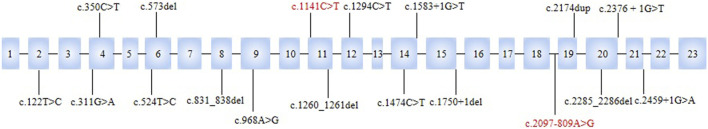
Locations of variants in *FIG4* associated with YVS. c.2097-809A>G and c.1141C>T were identified in the present study.

RT-PCR of *FIG4* exons 16 to 20 in mRNAs from the patient displayed an aberrant splicing transcript of 579 bp in addition to a 451-bp wild-type product (Figure 3A). Afterward, TA cloning was carried out, and cDNA sequencing results showed that the longer abnormal transcript comprised a 128-bp pseudo-exon from intron 18 (*FIG4*: c.2097-808_ 2097-681) (Figures 3B, C), resulting in a frameshift and generating a premature termination codon at residue 822 (p.Asp700Valfs*123).

### 3.4 Prenatal diagnosis of the *FIG4* gene

In 2024, the mother was pregnant with her second child. We employed Sanger sequencing for amniotic fluid sample prenatal testing and identified that the fetus also unfortunately had compound heterozygous variants of c.2097-809A>G and c.1141C>T in the *FIG4* gene (Figure 2B). Prenatal ultrasound showed a low conus medullaris position and an expanded right lateral ventricle (Figure 1C). Furthermore, SNP-array results showed that it was a female fetus. After genetic counseling, the couple chose to abort the child. After obtaining consent from the parents, we observed some phenotypes in the aborted fetus, such as low ear position and dysplasia of thumbs and halluces (Figure 1D). The parents may undergo pre-implantation genetic testing for monogenic disorders (PGT-M) for the next child.

## 4 Discussion

In the era of rapid sequencing technology, whole-genome sequencing has become a powerful tool for identifying candidate variants and enabling appropriate diagnoses—especially when pathogenic variants are concealed in patients with the typical phenotype and cannot be detected by routine exonic DNA sequencing methods such as Sanger sequencing or whole-exome sequencing. In this study, we detected a compound heterozygous variant in *FIG4*: c.1141C>T (p.R381*) and c.2097-809A>G by WGS. c.2097-809A>G is the first deep intronic variant in the *FIG4* gene associated with Yunis–Varon syndrome.

Yunis–Varón syndrome is caused by complete loss-of-function of *FIG4* and is characterized by severe neurological anomalies, cleidocranial dysplasia, and digital anomalies (Campeau et al., 2013). Other frequently reported malformations include a wide fontanelle with diastasis of cranial sutures, sparse scalp hairs, microcephaly, ectodermal malformations, bilateral hip dislocation, genital abnormalities, and central nervous system abnormalities such as Dandy–Walker malformation, agenesis of the corpus callosum, hydrocephalus, and hypoplastic vermis (Umair et al., 2021). Cardiovascular abnormalities such as ventricular hypertrophy and tetralogy of Fallot have also been reported (Adès et al., 1993; Abdul Wahab Siddique et al., 2019). The dysmorphic features observed in our three patients were consistent with those reported previously.

Introns are very important for eukaryotic evolution. The identification of genes with correct exon–intron architecture plays an important role in eukaryotic genome annotation (Mishra et al., 2021), and alternative splicing is crucial for the enhancement of transcriptome and proteome diversity (Keren et al., 2010). Nowadays, WGS has greatly improved the diagnosis of genetic diseases and increased the number of pathogenic deep intronic variants. These deleterious deep intronic variants usually generate pseudo-exon inclusion by the activation of atypical splice sites or changes in splicing regulatory elements (Vaz-Drago et al., 2017).

In the present study, we identified two variants in *FIG4*: c.1141C>T (p.R381*) inherited from the mother and c.2097-809A>G inherited from the father in the proband. His affected brother had a similar phenotype, but unfortunately, we could not obtain his sample to confirm the molecular results. c.1141C>T causes a premature termination codon at position 381 and has been reported in two children with early-onset CMT4J. c.2097-809A>G may destroy the acceptor sites predicted by SpliceAI. Subsequently, RT-PCR of *FIG4* in the patient’s mRNAs displays an aberrant splicing transcript of 579 bp compared to a 451-bp wild-type product (Figure 3A) that hinted at a pseudo-exon inclusion in *FIG4*. We identified a 128-bp pseudo-exon from intron 18 (*FIG4*: c.2097-808_ 2097-681) in the mutant type by RT-PCR analysis and TA cloning. We put the full sequence of *FIG4* containing the pseudo-exon into EditSeq software, and the translated protein presented a frameshift and resulted in a premature termination codon at residue 822 (p.Asp700Valfs*123). p.Asp700Valfs*123 is predicted to cause loss-of-function of the protein as a frameshift truncating mutation, resulting in an altered protein that lacks the C-terminal residues of the wild-type FIG4 protein responsible for membrane anchoring (Campeau et al., 2013). Nonsense-mediated mRNA decay is likely to occur because the variants remove more than 10% of the protein. After 1 year, the mother was pregnant again. Prenatal diagnosis showed that her second child also had compound heterozygous variants in *FIG4*. In addition, the aborted fetus exhibited a low conus medullaris position, an expanded right lateral ventricle, a low ear position, and dysplasia of thumbs and halluces. Unfortunately, the family had another YVS patient because of c.2097-809A>G and c.1141C>T in the *FIG4* gene. According to the ACMG variant classification guideline, c.1141C>T could be classified as pathogenic (PVS1, PM3, PM2_Supporting, PP1, and PP4), and c.2097-809A>G could also be classified as pathogenic (PVS1, PM2_Supporting, PM3, PP1, and PP4). Notably, c.2097-809A>G appeared to be the first deep intronic variant in the *FIG4* gene associated with YVS (Figure 4).

To date, 18 variants of YVS have been described in previous publications and are shown in Figure 4. Of them, 61.1% (11 of 18) are loss-of-function variants, including 1 nonsense variant, 5 frameshift variants, and 5 splicing variants. c.122T>C p.(Ile41Thr), c.1474C>T p.(Arg492Cys), c.968A>G p.(Gln323Arg), c.311G>A(p.Gly104Asp), and c.524T>C alter highly conserved amino acid residues located at important domains and are predicted to affect FIG4 function (Campeau et al., 2013; Umair et al., 2021; Beauregard-Lacroix et al., 2024). In those cases, 10 patients had prenatal ultrasound manifestations (Table 1). The most frequent clinical feature in fetuses (60%, 6 of 10) is structural brain anomalies such as dilated ventricles and Dandy–Walker malformation rather than limb abnormalities (>75% of cases) (Beauregard-Lacroix et al., 2024). Only two children (20%, 2 of 10) had absent thumbs or halluces because of a low prenatal detection rate in prenatal evaluation (Vij et al., 2023). The affected individuals also presented with other manifestations, such as fetal pyelectasis, fetal hydrops, and growth retardation. It is worth noting that two patients in our study had abnormalities that have not been previously reported: a posterior fossa cyst and a low conus medullaris position with coccyx curvature abnormality. All of the cases were found in the third trimester, except one in the second trimester, which demonstrates a significant barrier to early diagnosis and intervention for patients’ families.

**TABLE 1:** Summary of prenatal ultrasound clinical findings in YVS.

Reference	Familial relationship	Variant	Gender	Postnatal evolution	Prenatal ultrasound finding
Wang et al. (2023)	One affected child	c.350C>T and c.2376 + 1G>T	NA	19W	A dilated ventricle and fetal pyelectasis
Peng et al. (2021)	One affected child	c.573del, and c.2174dup	NA	24W	Scaphocephaly, absence of bilateral thumbs, hypospadias, pericardial effusion, and umbilical cord cyst
Walch et al. (2000)	One affected child	NA	F	NA	Hydrocephalus with Dandy–Walker malformation
Nakajima et al. (2013)	One affected child	c.1750+1del and c.2285_2286del	F	NA	Intrauterine growth retardation
Sumi et al. (2004)	One affected child	NA	M	30W	Fetal growth retardation and microcephaly
Basel-Vanagaite et al. (2008)	One affected child	NA	F	37W	Fetal hydrops and polyhydramnios
Corona-Rivera et al. (2011)	Two affected siblings	NA	F	NA	NA
		NA	F	29W	Absent thumbs and big toes
Umair et al. (2021)	One affected child	c.968A>G	M	35W	Severe oligohydramnios
This study	Two affected siblings	NA	M	24W	A dilated fourth ventricle, a posterior fossa cyst, low conus medullaris position with coccyx curvature abnormality, persistent left superior vena cava, slightly enlarged right heart, and a small amount of pericardial effusion
		c.1141C>T and c.2097-809A>G	M	NA	NA
		c.1141C>T and c.2097-809A>G	F	23W	Low conus medullaris position and an expanded right lateral ventricle

F, female; M, male; NA, not assessed.

Pathogenic deep intronic variants have been reported in more than 100 disease-associated genes. Given that the deep intronic variants identified in the present study could be detected using whole-genome sequencing and RT-PCR and splicing analysis are usually used to validate variants that have an effect on splicing in a molecular diagnostic setting (Vaz-Drago et al., 2017; Zhang et al., 2023), in this study, we used WGS to effectively detect deep intronic variants that have been widely employed in the diagnosis of genetic diseases. We detected the first deep intronic variant (c.2097-809A>G) in *FIG4.* Pathogenicity analysis of deep intronic variants in *FIG4* can improve the genetic diagnosis rate of YVS and highlight the importance of screening of non-coding regions with functional assays to decipher the hidden variant in YVS. At the same time, in this family, spinal deformity was observed as a new phenotype in YVS. However, the small sample size is quite a limitation to our study. Therefore, additional efforts on large cohort studies are urgently needed to better clarify the genotype–phenotype correlation and provide refined genetic counseling.

## 5 Conclusion

We detected the first deep intronic variant in *FIG4* (c.2097-809A>G) in a Chinese family with three neonates presenting the hallmark features of YVS, thereby linking this variant to the syndrome and expanding the unknown genetic variant spectrum. The splicing analysis shows how c.2097-809A>G influences the transcript and contributes to YVS. This study provides additional molecular and clinical information and broadens our understanding of the molecular mechanisms involved in the disease course.

## Data Availability

The datasets presented in this article are not readily available to assure patient confidentiality and participant privacy. Requests to access the datasets should be directed to xiangjingjing2013@163.com.
